# The Influence of COVID-19 on Retirement and Longevity Planning: A Multigenerational Perspective

**DOI:** 10.1093/geroni/igab046.312

**Published:** 2021-12-17

**Authors:** Julie Miller, Martina Raue, Lisa D'Ambrosio

**Affiliations:** 1 MIT, Cambridge, Massachusetts, United States; 2 MIT AgeLab, Cambridge, Massachusetts, United States; 3 Massachusetts Institute of Technology, Cambridge, Massachusetts, United States

## Abstract

For many, the COVID-19 pandemic has painted a new economic picture of longevity. Results from the series demonstrated that younger generations surveyed—Gen Xers, Millennials, and Zoomers— generally reported elevated levels of worry about how the pandemic would affect their long-term finances. Baby Boomers, by comparison, evinced significantly less worry. And, although the pandemic has had a disproportionate economic impact on lower-income individuals, a higher income did not appear to confer freedom from worry. When it comes to planning for longevity, worry can be paralyzing. Zoomers were the generation most likely to agree that the future is too uncertain for them to even think about planning financially for retirement, and this sentiment applied for other generations as well. This presentation will also describe other ways in which the COVID-19 pandemic had influenced respondents’ perceived longevity preparedness and attitudes across a variety of demographic and socio-economic characteristics.

